# Awareness and use of a free vapes programme for smoking cessation in England: findings from the 2024 ITC 4 Country Smoking and Vaping Survey

**DOI:** 10.1186/s12889-026-28769-0

**Published:** 2026-08-05

**Authors:** Martine Skumlien, Katherine A East, Erikas Simonavičius, Andrew Hyland, Geoffrey T Fong, Grace Li, Anne C K Quah, Christian Boudreau, Ann McNeill, Leonie Brose

**Affiliations:** 1 NIHR Policy Research Unit in Addictions, Department of Addictions, Institute of Psychiatry, Psychology & Neuroscience, King’s College London, London, UK; 2 Social, Genetic & Developmental Psychiatry Centre, Institute of Psychiatry, Psychology & Neuroscience, King’s College London, London, UK; 3 Department of Primary Care and Public Health, Brighton and Sussex Medical School, University of Brighton and University of Sussex, Brighton, UK; 4 European Union Drugs Agency, Lisbon, Portugal; 5 Roswell Park Comprehensive Cancer Centre, University of Buffalo, New York, USA; 6 Department of Psychology, University of Waterloo, Waterloo, ON Canada; 7 School of Public Health Sciences, University of Waterloo, Waterloo, ON Canada; 8 Ontario Institute for Cancer Research, Toronto, ON Canada; 9 Department of Statistics and Actuarial Science, University of Waterloo, Waterloo, ON Canada

**Keywords:** Vaping, Smoking cessation, Swap to Stop, Vaping harm perception

## Abstract

**Background:**

England’s ‘Swap to Stop’ programme provides local authorities with free vape starter kits for adults who smoke. This study aimed to evaluate the reach, uptake, and correlates of engagement with Swap to Stop within the first year after the start of implementation.

**Methods:**

Our sample were adults (*n* = 2009) who currently smoke or recently stopped smoking from the England arm of the 2024 International Tobacco Control Four Country Smoking and Vaping Survey. We calculated weighted proportions for Swap to Stop awareness, offer, interest, and participation, and reasons for no interest/participation. We performed weighted binary logistic regressions to explore associations between sociodemographics, health, smoking cessation attempts, and vaping status and Swap to Stop awareness, offer, and interest/participation, and between interest/participation and vaping harm perceptions.

**Results:**

Overall, 11.0% had heard of Swap to Stop, of whom 43.6% were offered to take part. Among those offered, 63.2% had participated; among those not offered, 55.1% were interested. The most common reasons for no interest/participation were not being interested in vapes (60.7%) and believing them to be unsafe (57.5%) or no less harmful than smoking (56.6%). Awareness was higher among non-White than White participants (AOR = 1.86), those with high (AOR = 1.78) and medium (AOR = 1.89) than lower incomes, and those who did than did not currently vape (AOR = 3.44), and lower among women than men (AOR = 0.57) and those aged ≥ 40 than ≤ 24 years (AOR = 0.28) and 25–39 years (AOR = 0.34). Offer of participation was also lower among those aged ≥ 40 years (AOR = 0.06 vs. ≤24 years, AOR = 0.21 vs. 25–39 years). Interest/participation was higher among people with than without prior smoking cessation attempts (AOR = 2.52) and among people who were than were not currently vaping (AOR = 4.50), and lower among women than men (AOR = 0.32). Interest/participation was associated with perceiving vaping as less harmful than smoking (AOR = 2.61).

**Conclusion:**

Within its first year, across England, awareness of Swap to Stop was limited, though uptake and interest in the programme were high. Awareness may have been higher in areas that adopted the programme early and may increase with maturation. Targeted outreach and communication on the relative harms of vaping is key to maximising public health impact.

**Supplementary Information:**

The online version contains supplementary material available at https://doi.org/10.1186/s12889-026-28769-0.

## Introduction

Tobacco smoking is one of the leading causes of preventable morbidity, mortality, and health inequalities in England [1–5]. Around 5.3 million people in the UK aged ≥ 18 smoked cigarettes in 2024, corresponding to 10.6% of the population [6], and this number likely underestimates the true prevalence [7]. In 2019, the UK government set a smokefree target for England, defined as a smoking prevalence of < 5% by 2030 [8, 9]. In 2022, an independent review concluded that England would miss this target by at least seven years, and by 14 years in the most socioeconomically deprived areas, unless innovative approaches were implemented [10]. One such approach is harnessing nicotine vaping for smoking cessation.

Nicotine vaping is the most popular cessation aid among people who try to quit smoking in England [11]. Vaping products deliver nicotine while exposing those who use them to fewer harmful chemicals than cigarettes. There is high-certainty evidence that nicotine vapes increase quit rates compared to licensed nicotine replacement therapy [12, 13], and they have been found to be cost effective [14, 15]. One survey study found that nicotine vapes may have helped 30,000 to 50,000 additional persons who smoke in England to successfully quit each year since 2013 [16].

Based on this evidence, the Swap to Stop programme was announced in England in April 2023 [17]. England’s Swap to Stop is a world-first programme which provides free vape starter kits alongside behavioural support to adults who want to quit smoking. It aims to enhance local services and is delivered by local authorities or partnerships who can apply for allocations of vouchers or vapes, choosing from a range of vape models and liquids (excluding disposable vapes). Implementation started in December 2023, with the first vapes distributed around March 2024, and uptake increasing throughout that year.

The Swap to Stop programme aims to target populations with a relatively higher smoking prevalence and who have greater risk of smoking-related harms, including people from more disadvantaged socioeconomic groups, people with long-term physical or mental health conditions, and those for whom conventional cessation methods have been ineffective. Reaching these groups is critical to ensuring the continuing decline in smoking prevalence in England. The overall goal of the Swap to Stop programme is to distribute vapes to ~ 1 in 5 people who smoke in England [17]. This national programme could also help rectify incorrect perceptions that vaping is equally as or more harmful than smoking, which is a key barrier to switching from smoking to vaping [18–20].

As part of a wider evaluation [21], the current study aimed to estimate the reach, uptake, and correlates of engagement with the Swap to Stop programme using data from the 2024 International Tobacco Control 4 Country Smoking and Vaping (ITC 4CV) survey. We had four research questions:

1. What proportion of people in England who smoke or recently quit smoking are aware of, have been offered, are interested in, and have participated in the Swap to Stop programme?
2. Which reasons do people who smoke or recently quit smoking give for not wanting to participate in Swap to Stop?
3. Which sociodemographic, health, smoking, and vaping characteristics are associated with being aware of, being offered, being interested in, and having participated in Swap to Stop?
4. Is Swap to Stop participation associated with vaping harm perceptions?

We also explored additional Swap to Stop outcomes collected in the ITC study, including the number of people who found participation helpful, were still using the same type of vape they were given, and had received additional support as part of the programme.

Our pre-registered [22] hypotheses were as follows:

1. Previous attempts to stop smoking and heavier smoking will be associated with being aware of, being offered, being interested in, and having participated in Swap to Stop.
2. Participation in Swap to Stop will be associated with perceiving vaping as less harmful than smoking.

Although Swap to Stop was targeted at those with lower income and worse physical and mental health, we did not have clear predictions for these variables due to the inverse care law [23], which states that the availability of healthcare varies inversely with the population(s) most in need.

## Methods

### Pre-registration

Research questions and analyses were pre-registered to the Open Science Framework [22]. Deviations from the pre-registration are reported in the Supplemental Materials. Briefly, we collapsed response categories for gender, ethnicity, income, and vaping status due to there being too few participants in some of the categories. We also did not conduct planned analyses with smoking status as a predictor, due to this interacting significantly with several other predictors.

### Design, setting, and sample

This is a cross-sectional study using England data from the fifth wave of the ITC 4CV (ITC 4CV5) survey, collected between July and November 2024. Full details on ITC methods are available in the technical report [24]. Briefly, adults aged ≥ 18 years who currently smoke (at least monthly), recently quit smoking (within the last 5 years), or who currently use a vape (at least monthly), heated tobacco product, snus, or tobacco-free oral nicotine product were recruited from Rakuten Insight and IPSOS UK online panels and from the pool of participants who had completed previous ITC waves. The present analyses included those who were currently smoking daily, weekly, monthly or less than monthly, or had quit smoking in the past 12 months (in an attempt to exclude participants who quit before Swap to Stop was launched).

The total sample with valid responses from England for ITC 4CV5 was 2287 participants. After excluding participants who did not currently smoke or had previously smoked but quit > 12 months ago (*n* = 266) and participants who did not answer the Swap to Stop questions (*n* = 12), we were left with a final sample of *n* = 2009.

All participants gave informed consent to taking part in the study. The study received ethical approval from the University of Waterloo Office of Research Ethics (REB#20803/30570) and the King’s College London Research Ethics Office (IRB RESCM-17/18-2240) and was conducted in accordance with the Declaration of Helsinki.

### Measures

A full summary of variable measurement and manipulation, including any deviations from the pre-registered analysis protocol, is provided in the Supplemental Methods and Supplemental Table 1. All variables were selected a priori based on the target groups for Swap to Stop.

Participants who responded “don’t know” were always included with the reference category. Participants who refused to answer any question used in the analysis were excluded. Below, we describe how variables were defined for logistic regression models.

#### Sociodemographics

Age Group: 18–24 years, 25–39 years, ≥ 40 years.

Gender: Man, Woman/Other.

Ethnicity: White, any other race/ethnicity.

Income: ≤£30,000, £30,001-£50,000, ≥£50,001.

For all four variables, the first category listed was the reference category.

#### Health

Mental Health Diagnosis: Ever diagnosed with a mental health condition: yes, no.

Physical Health Diagnosis: Ever diagnosed with diabetes, heart disease, cancer, asthma, emphysema, or chronic bronchitis: yes, no.

For both, ‘no’ was treated as the reference category.

#### Smoking and vaping

Past-Year Daily Smoking: Currently smoking daily or smoked daily in the past year: yes, no.

Prior Cessation Attempt: Ever made an attempt to stop smoking: yes, no.

Vaping Status: Currently vape daily/weekly/monthly/less than monthly): yes, no.

For all, ‘no’ was treated as the reference category.

#### Outcomes

Outcomes were (i) being aware of Swap-to-Stop (‘Awareness’); (ii) having been offered Swap to Stop, among those aware (‘Offer’), (iii) having participated in Swap to Stop, among those having been offered to take part (‘Participation’); (iv) being interested in Swap to Stop, among those not having been offered to take part (‘Interest’); (v) reasons for not participating in Swap to Stop among those who reported that they were not interested in taking part or who had been offered to take part but declined; and (iv) perceiving vaping as less harmful than smoking (‘Vaping Harm Perception’). All were defined as having yes/no response options, with ‘no/don’t know’ treated as the reference category. For the logistic regressions, Swap to Stop Interest and Participation were collapsed into one variable (‘Interest/Participation’) due to the low sample size resulting from splitting this variable. Additional Swap to Stop variables are reported in Supplemental Table 1.

### Statistical analyses

Analyses were conducted in R 4.4.1 using the ‘survey’ package [25]. All analyses were weighted using sampling weights computed with a raking algorithm based on smoking/vaping status, geographic region, and demographic variables [24]. Participants who refused to provide a response for any one variable were excluded from the relevant model (i.e., listwise).

We calculated weighted proportions and 95% confidence intervals (CIs) for Swap to Stop Awareness, Offer, Interest, and Participation to address research question 1, and reasons for not participating or being interested in Swap to Stop to address research question 2. To explore whether engagement with Swap to Stop was higher among target groups (research question 3), we ran weighted binary logistic regressions, separately regressing Swap to Stop Awareness, Offer, and Interest/Participation onto all sociodemographic, health, smoking, and vaping measures. To address whether Swap to Stop participation was associated with perceiving vaping as less harmful than smoking (research question 4), we ran weighted binary logistic regressions, regressing vaping harm perceptions onto all sociodemographic, health, smoking, and vaping variables plus Swap to Stop Interest/Participation. We also repeated this model with Awareness instead of Interest/Participation as the predictor. Finally, we repeated all logistic regression models without weights, to assess how survey weighting impacted the findings. We did not include any additional confounders in our models.

## Results

### Participant characteristics

Participant characteristics are provided in Table 1. The majority of participants were aged ≥ 40 years (61%), men (51%), White (85%), had no mental (67%) or physical (76%) health diagnoses, and currently smoked cigarettes daily (66%) and/or had smoked daily in the past 12 months (83%) (weighted percentages). Most had previously attempted to quit smoking (70%). More participants were currently vaping (*n* = 1167) than not (*n* = 842), but this reversed with weighting (32% currently vaping).

**Table 1:** Participant characteristics

	All participants (*n* = 2009)		Participants aware of Swap to Stop (*n* = 430)	
	*n*	% (weighted)	*n*	% (weighted)
Age Group				
≤ 24 years	191	10.5	96	19.0
25–39 years	587	28.4	228	46.8
≥ 40 years	1231	61.1	106	34.2
Gender				
Man	1102	51.2	276	57.4
Woman	900	48.0	153	42.5
Other	8	0.9	1	0.1
Ethnicity				
White	1743	85.4	356	73.2
Mixed/multiple	60	3.3	19	5.6
Asian	90	4.8	22	8.2
Black	92	3.9	30	10.4
Other	4	0.6	1	1.5
Don’t know	2	0.0	1	0.4
Refused	18	1.9	1	0.8
Income				
≤£30,000	822	47.9	129	35.9
£30,001 - £50,000	474	22.5	109	30.4
≥£50,001	599	20.3	185	31.4
Don’t know	14	1.0	2	0.7
Refused	100	8.3	5	1.7
Mental Health Diagnosis				
No	1449	66.7	303	62.3
Yes	485	31.1	98	34.4
Don’t know	24	0.6	6	1.3
Refused	51	1.7	23	2.0
Physical Health Diagnosis				
No	1480	76.1	318	77.0
Yes	464	22.1	87	20.7
Don’t know	22	0.6	3	0.5
Refused	43	1.2	22	1.8
Smoking Status				
Quit smoking in the past 12 months	108	20.3	14	25.1
Currently smoking non-daily	391	14.1	95	21.6
Currently smoking daily	1510	65.6	321	53.3
Past-Year Daily Smoking				
No	308	16.8	67	29.0
Yes	1697	82.8	362	70.7
Refused/missing	4	0.4	1	0.2
Quit Attempt, ever				
No	671	29.0	128	28.5
Yes	1303	69.8	296	71.1
Don’t know	21	0.6	4	0.4
Refused/missing	14	0.6	2	0.1
Vaping Status				
Not currently vaping	842	68.2	50	37.5
Currently vaping daily/non-daily	1167	31.8	380	62.5
Vaping Harm Perception				
Much/somewhat more harmful	334	14.0	138	25.5
Equally harmful	595	33.9	91	27.2
Much/somewhat less harmful	818	36.8	186	40.3
Don’t know	241	13.3	12	4.8
Refused/missing	21	1.9	3	2.2
Aware of Swap to Stop				
No	1530	86.3		-
Yes	430	11.0		-
Don’t know	49	2.7		-
Offered Swap to Stop				
Yes, participated	-		161	27.6
Yes, but did not participate	-		109	16.0
No, but interested	-		115	31.0
No, and not interested	-		44	25.3
Don’t know	-		1	0.1

#### Research question 1. What proportion of people in England who smoke or recently quit smoking are aware of, have been offered, are interested in participating, and have participated in the Swap to Stop programme?

**Fig. 1:**
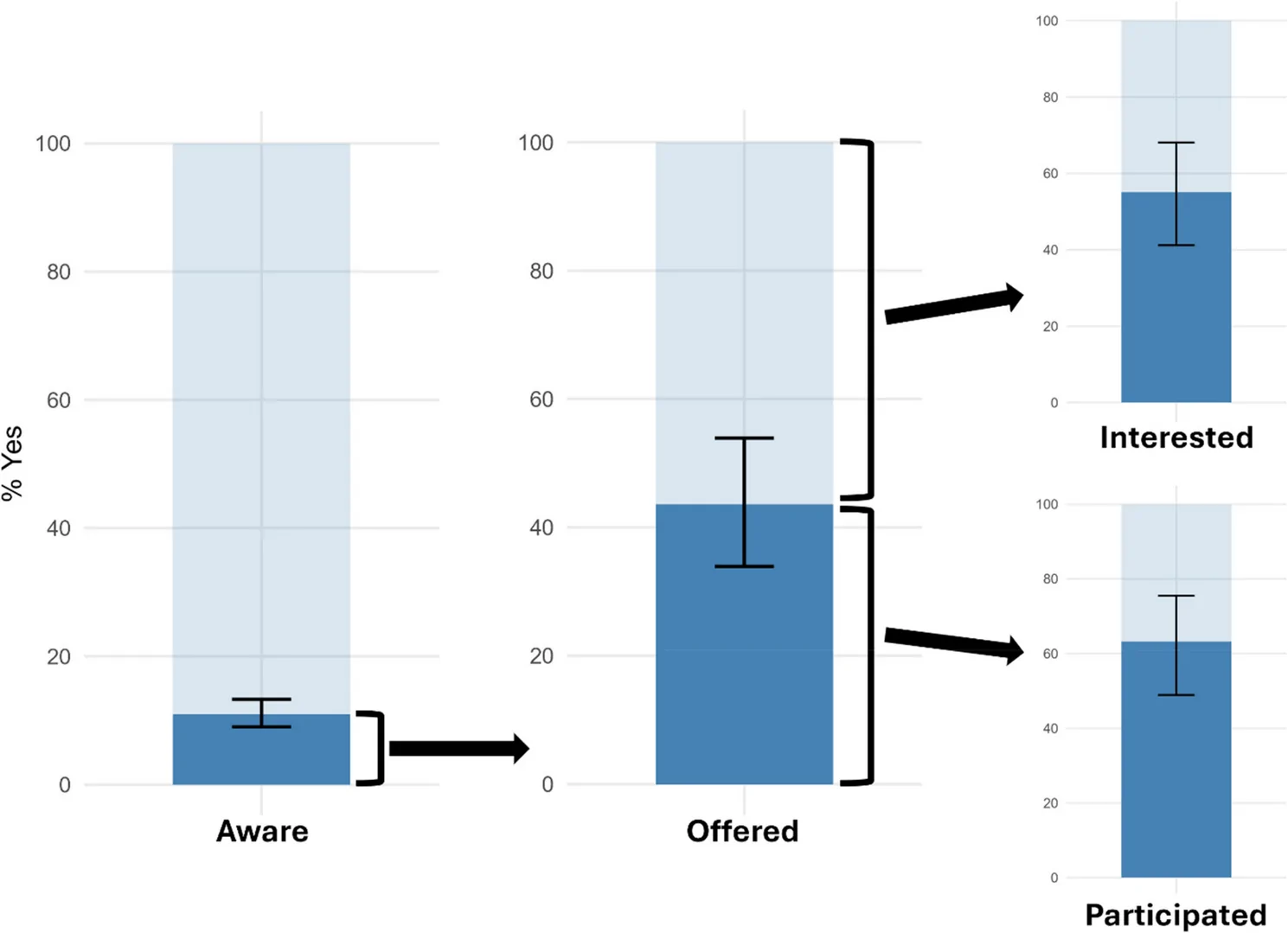
Proportion of participants who were aware of, offered, interested in, and participated in Swap to Stop (weighted % and confidence intervals)

The overall weighted percentage of participants who reported being aware of the Swap to Stop programme was 11% (95% CI, 9%-13%) (Fig. 1). Of those who were aware, 44% (34%-54%) had been offered to take part, of whom 63% (50%-77%) had participated. Of those who had not been offered to take part, 55% (41%-69%) were interested. Of those who took part, 96% (90%-99%) found the programme helpful, 65% (42%-89%) were still using the same type of vape they were given, and 69% (47%-92%) had received other support such as meetings, calls or an app in addition to the vape (Supplemental Table 2).

### Research question 2. Which reasons do people who smoke or recently quit smoking give for not wanting to participate in Swap to Stop?

The most common reasons for not being interested in or declining to participate were not being interested in using e-cigarettes (61%) and not believing e-cigarettes to be safe (58%), less harmful than smoking (57%), or helpful to quitting smoking (54%) (Table 2).

**Table 2:** Reasons for not being interested in or declining to participate in Swap to Stop

	*n*	% (weighted)	95% confidence interval (weighted)
I’m not interested in using e-cigarettes			
Yes	71	60.7	45.8–75.5
No/don’t know	78	39.3	24.5–54.2
I don’t believe e-cigarettes are safe			
Yes	60	57.5	42.4–72.6
No/don’t know	90	42.5	27.4–57.6
I don’t believe e-cigarettes are less harmful than smoking			
Yes	78	56.6	41.6–71.5
No/don’t know	71	43.4	28.5–58.4
I don’t believe e-cigarettes will help me stop smoking			
Yes	83	53.7	38.4–69.0
No/don’t know	69	46.3	31.0–61.6
I can just buy e-cigarettes myself			
Yes	84	46.7	31.6–61.8
No/don’t know	65	53.3	38.2–68.4
I don’t want support			
Yes	66	42.1	26.7–57.6
No/don’t know	81	57.9	42.4–73.3
I didn’t like the e-cigarette(s) being offered			
Yes	61	33.2	19.6–46.9
No/don’t know	89	66.8	53.1–80.4
I’m not interested in stopping smoking			
Yes	48	27.6	13.6–41.6
No/don’t know	101	72.4	58.4–86.4
There was a long waiting list			
Yes	45	25.2	11.4–38.9
No/don’t know	105	74.8	61.1–88.6
Other			
Yes	12	12.3	2.8–21.7
No/don’t know	125	87.7	78.3–97.2

Sample sizes vary because different numbers of participants did not provide answers for each item

#### Research question 3. Which sociodemographic, health, smoking, and vaping characteristics are associated with being aware of, being offered, and being interested in/having participated in Swap to Stop?

A total of 1844 participants had valid data for all variables in the logistic regression for Awareness and 396 participants had valid data for all variables in the logistic regressions for Offer and Interest/Participation. Results of all models are presented in Table 3.

**Table 3:** Results of weighted binary multivariable logistic regression models for Swap to Stop outcomes

	Swap to Stop Awareness^a^				Swap to Stop Offer^b^				Swap to Stop Interest/Participation^b^		
	*n* in model	*n* ‘yes’ for outcome	AOR (95% CI)	*p*	*n* in model	*n* ‘yes’ for outcome	AOR (95% CI)	*p*	*n* ‘yes’ for outcome	AOR (95% CI)	*p*
Age Group											
≤ 24 years (ref)	158	77	-	-	77	67	-	-	59	-	-
25–39 years	554	217	0.81 (0.35–1.91)	0.63	217	143	0.29 (0.07–1.13)	0.07	130	0.34 (0.10–1.17)	0.09
≥ 40 years	1132	102	0.28 (0.12–0.65)	0.003	102	32	0.06 (0.02–0.23)	< 0.001	63	0.69 (0.19–2.46)	0.56
Gender											
Man (ref)	1019	255	-	-	255	164	-	-	163	-	-
Woman or other	825	141	0.57 (0.33–0.97)	0.04	141	78	0.60 (0.22–1.62)	0.31	89	0.32 (0.15–0.69)	0.004
Ethnicity											
White (ref)	1612	327	-	-	327	210	-	-	211	-	-
Any other race/ethnicity	232	69	1.86 (1.02–3.39)	0.04	69	32	0.88 (0.28–2.76)	0.82	41	1.34 (0.52–3.48)	0.55
Income											
≤£30,000 (ref)	799	114	-	-	114	77	-	-	70	-	-
£30,001 - £50,000	453	100	1.89 (1.04–3.44)	0.04	100	64	0.84 (0.28–2.55)	0.75	67	1.77 (0.65–4.80)	0.26
≥£50,001	592	182	1.78 (1.00–3.17)	0.05	182	101	1.57 (0.47–5.24)	0.47	115	2.05 (0.71–5.95)	0.19
Mental Health Diagnosis											
No (ref)	1375	298	-	-	298	178	-	-	191	-	-
Yes	469	98	1.34 (0.79–2.27)	0.27	98	64	1.21 (0.35–4.20)	0.77	61	1.43 (0.52–3.91)	0.49
Physical Health Diagnosis											
No (ref)	1418	313	-	-	313	179	-	-	197	-	-
Yes	426	83	1.44 (0.80–2.60)	0.23	83	63	0.85 (0.24–2.98)	0.80	55	0.62 (0.22–1.77)	0.37
Past-Year Daily Smoking											
No (ref)	283	65	-	-	65	30	-	-	39	-	-
Yes	1561	331	0.61 (0.33–1.15)	0.13	331	212	1.88 (0.53–6.65)	0.33	213	0.64 (0.21–1.89)	0.41
Quit Attempts											
No (ref)	657	128	-	-	128	73	-	-	71	-	-
Yes	1187	268	1.20 (0.70–2.06)	0.50	268	169	1.34 (0.47–3.83)	0.59	181	2.52 (0.99–6.44)	0.05
Vaping Status											
Not currently vaping (ref)	754	42	-	-	42	7	-	-	16	-	-
Currently vaping	1090	354	3.44 (2.11–5.62)	< 0.001	354	235	2.74 (0.90–8.34)	0.08	236	4.50 (1.66–12.19)	0.003

A Wald 2 d.f. test showed that the overall effect of Age Group was significant for Awareness (*F* = 9.72, *p* < .001) and Offer (*F* = 10.17, *p* < .001) but not for Interest/Participation (*F* = 1.75, *p* = .17). Odds ratios for the Age Group comparison ≥40 years versus 25-39 years were 0.34 (0.20 – 0.58) for Awareness and 0.21 (0.08 – 0.56) for Offer. A Wald 2 d.f. test showed that the overall effect of Income was marginally significant for Awareness (*F* = 2.98, *p* = .051) but not significant for Offer (*F* = 0.54, *p* = .59), or Interest/Participation (*F* = 1.07, *p* = .34). Odds ratios for the Income comparison ≥£51,001 versus £30,001-£50,000 was 0.94 (0.49 – 1.82) for Awareness.

Abbreviations: *AOR* adjusted odds ratio, *CI* confidence interval, *ref* reference category

^a^*n* = 1844

^b^*n* = 396

Awareness was associated with being aged below 40 years, being a man, non-White ethnicity, earning more than £30,000, and currently vaping. Mental or physical health diagnosis, past-year daily smoking, and prior cessation attempt were not significantly associated with awareness. Offer was associated with with being aged below 40 years but no other variables. Interest/participation was associated with being a man, having made a prior quit attempt, and currently vaping.

Results of the unweighted analyses are presented in Supplemental Table 3. In these models, the association between Ethnicity and Awareness and the association between Gender and Interest/Participation were no longer significant. However, several factors that were not significant in the weighted models were significant in the unweighted models. Specifically, Awareness was additionally associated with being ≤ 24 years vs. 25–39 years, having smoked daily in the past year, and having ever made a cessation attempt; Offer was additionally associated with being ≤ 24 years vs. 25–39 years, White ethnicity, having a physical health diagnosis, and currently vaping; and Interest/Participation was additionally associated with being ≤ 24 years vs. 25–39 years.

#### Research question 4. Is Swap to Stop participation associated with vaping harm perceptions?

Overall, 38% (34%-41%) believed vaping to be less harmful than smoking. Interest/Participation, but not Awareness, was associated with higher odds of believing vaping to be less harmful than smoking (Table 4). These associations remained similar in the unweighted models (Supplemental Table 3).

**Table 4:** Results of weighted binary logistic regression models for vaping harm perception

	Perceiving vaping to be less harmful than smoking^a^				Perceiving vaping to be less harmful than smoking, among those aware of Swap to Stop^b^			
	*n* in model	*n* ‘yes’ for outcome	AOR (95% CI)	*p*	*n* in model	*n* ‘yes’ for outcome	AOR (95% CI)	*p*
Age-Group								
≤ 24 years (ref)	158	58	-	-	77	35	-	-
25–39 years	550	229	0.97 (0.44–2.15)	0.93	215	99	0.68 (0.22–2.05)	0.49
≥ 40 years	1121	483	1.76 (0.86–3.63)	0.12	101	48	0.87 (0.23–3.24)	0.83
Gender								
Man (ref)	1012	452	-	-	254	108	-	-
Woman or other	817	318	0.64 (0.47–0.87)	.01	139	74	1.41 (0.66–3.03)	0.38
Ethnicity								
White (ref)	1599	665	-	-	325	143	-	-
Any other race/ethnicity	230	105	1.80 (1.05–3.11)	.03	68	39	2.71 (0.96–7.61)	0.06
Income								
≤£30,000 (ref)	792	276	-	-	113	25	-	-
£30,001 - £50,000	449	211	1.64 (1.13–2.39)	.01	99	59	3.14 (1.19–8.32)	.02
≥£50,001	588	283	1.32 (0.87–2.00)	0.19	181	98	2.29 (0.82–6.42)	0.11
Mental Health								
No (ref)	1362	579	-	-	296	153	-	-
Yes	467	191	1.24 (0.88–1.76)	0.22	97	29	0.81 (0.36–1.80)	0.60
Physical Health								
No (ref)	1410	611	-	-	311	160	-	-
Yes	419	159	0.83 (0.60–1.15)	0.27	82	22	0.95 (0.35–2.57)	0.92
Past-Year Daily Smoking								
No (ref)	282	128	-	-	64	37	-	-
Yes	1547	642	1.11 (0.68–1.81)	0.69	329	145	0.45 (0.15–1.34)	0.15
Quit Attempts								
No (ref)	651	230	-	-	127	54	-	-
Yes	1178	540	1.46 (1.04–2.05)	.03	266	128	4.73 (1.98–11.33)	< 0.001
Vaping Status								
Not currently vaping (ref)	742	227	-	-	42	10	-	-
Currently vaping	1087	543	2.48 (1.79–3.42)	< 0.001	351	172	2.00 (0.75–5.30)	0.17
Swap to Stop awareness								
No (ref)	1436	588	-	-	-	-	-	-
Yes	393	182	1.00 (0.60–1.67)	0.99	-	-	-	-
Swap to Stop interest/ participation								
No (ref)	-	-	-	-	144	53	-	-
Yes	-	-	-	-	249	129	2.61 (1.18–5.77)	0.02

A Wald 2 d.f. test showed that the overall effect of Age Group (*F *= 4.04, *p* = .02) and Income (*F* = 3.46, *p* = .03) were significant for the model with awareness. The odds ratio for the Age Group comparison ≥40 years versus 25-39 years was 1.82 (1.16 – 2.87) and the odds ratio for the Income comparison ≥£51,001 versus £30,001-£50,000 was 0.81 (0.51 – 1.26). A Wald 2 d.f. test showed that the overall effect of Age Group (*F* = 0.33, *p* = .72) and Income (*F* = 2.72, *p* = .07) were not significant for the model with interest/participation.

Abbreviations: *AOR* adjusted odds ratio, *CI* confidence interval, *ref * reference category

^a^*n* = 1829

^b^*n* = 393

## Discussion

This study explored the reach, uptake, and correlates of engagement with the Swap to Stop programme in England using cross-sectional data from the ITC study. Having ever attempted to quit smoking was associated with Swap to Stop interest/participation, as hypothesised, but not with being aware of or offered Swap to Stop. Swap to Stop interest/participation was associated with perceiving vaping as less harmful than smoking, also consistent with our hypotheses. These factors were additionally significant in our unweighted models, strengthening our conclusions. However, our hypothesis that heavier smoking would be associated with Swap to Stop awareness, offer, interest, and participation, was not supported. We also did not find that engagement was higher among those with lower income or worse mental or physical health, which the programme aims to reach.

We found a lower proportion of participants who were aware of Swap to Stop (11%) compared with another recent nationally representative survey (24%) [26]. The reason for this discrepancy is unclear but may in part be due to differences in sampling and weighting. Nonetheless, both studies found that older participants were less likely to have heard of Swap to Stop compared with younger participants. Indeed, in our study, the oldest age group had roughly 3x lower odds of being aware of Swap to Stop and 16x lower odds of being offered Swap to Stop compared with the youngest age group, corresponding to a very large effect size. Crucially, participants in the older age group were no less likely to say they were interested in or had participated in Swap to Stop. Therefore, guidance provided to services that offer vapes should emphasise that participants of any age may be interested in and benefit from taking part.

There are several reasons why awareness of Swap to Stop was relatively low. First, the current data were collected over a four-month period in mid-to-late 2024, but many local authorities did not fully develop their intervention until 2025. Moreover, one recent study which explored Swap to Stop expressions of interest by local authorities, found that uptake varied significantly across different parts of England [27]. Consequently, awareness may have been higher in regions with greater or earlier uptake, which the current national evaluation was not able to capture. Another important factor is the relative lack of publicity of Swap to Stop, which relied primarily on local promotion by stop smoking services rather than mass media campaigns. The absence of national branding may also have resulted in some participants not having badged the intervention as Swap to Stop, leading us to potentially underestimate awareness. National public-facing promotion, which also targets groups with significantly lower awareness including older adults, women, and people in lower income brackets, might be necessary to increase uptake.

Importantly, interest and participation in the programme among those who were aware were high (both > 50%) and almost everyone (96%) who took part reported finding it helpful. Moreover, another recent study found that the introduction of Swap to Stop was associated with a statistically significant increase in the number of quit attempts using vapes in England [28]. Therefore, despite low awareness, there is preliminary evidence that Swap to Stop has been successful in promoting stop smoking behaviours. However, future research is needed to evaluate the effectiveness of the programme in increasing smoking cessation, which was beyond the scope of the current study.

As hypothesised, having previously tried to quit smoking increased the odds of Swap to Stop interest/participation, suggesting that the programme might appeal more to those who have unsuccessfully tried other quitting methods. However, there was little evidence that the programme had disproportionately reached those most at risk of smoking harms. Indeed, there were no relationships between interest/participation and ethnicity, income, mental health, physical health, or past-year daily smoking, despite evidence that these groups were targeted by local authorities applying for the programme [27]. One caveat is that population surveys, including ITC, tend to underrepresent more disadvantaged groups of individuals [7]. Therefore, the current study may have underestimated the extent to which Swap to Stop so far has reached vulnerable groups.

Swap to Stop interest/participation was associated with almost threefold higher odds of perceiving vaping as less harmful than smoking, consistent with our hypothesis. As this is a cross-sectional study, we cannot conclude whether this was because believing that vaping is less harmful increased the likelihood of taking part, or whether taking part in Swap to Stop decreased vaping harm perceptions. However, previous studies have found that inaccurate harm perceptions are a significant barrier to switching from smoking to vaping [18–20], which we also found in the current study, and may be driven by conflicting public health information and negative media stories about vaping [29, 30]. Therefore, clear public health guidance and communication on the relative harms of smoking and vaping, for instance through psychoeducation [18, 31], should remain a priority.

A major strength of this study was the large and weighted nationally representative sample of people who smoke and recently quit smoking in England. Despite this, due to the relatively low number of participants that were aware of Swap to Stop, we had to collapse predictor categories for gender, ethnicity, income, and vaping status for logistic regressions. Future studies should explore these factors with greater precision. This is especially important for gender and ethnicity, where crude categories that do not distinguish non-binary from male/female gender identities, and Asian, Black, Mixed, and other ethnicities, cannot capture meaningful differences between these groups [32–35]. Additionally, the current study defined heavy smoking as having smoked daily in the past year, and other ways of measuring smoking severity, such as cigarettes per day, may yield different results. Moreover, the previous quit attempts variable did not distinguish between those who had tried to quit as part of Swap to Stop and those who had tried to quit through other means, which might have inflated the association between this variable and Swap to Stop interest or participation. Finally, the current study relied on self-report, which means that our ability to test associations was subject to participants being able to recall whether they had heard of the programme and could be influenced by social desirability bias, particularly for smoking and vaping behaviours.

This study suggests that the Swap to Stop programme achieved limited reach at the national level seven to 11 months after implementation, largely due to low awareness. However, participation among those offered the programme was high and almost all who took part found the programme helpful. Importantly, inaccurate vaping harm perceptions were a major barrier to uptake. National campaigns to widen the reach of the programme and clear communication on the relative harms of vaping versus smoking may be required to maximise the impact of Swap to Stop.

## Supplementary Information

Supplementary Material 1.

## Data Availability

The data supporting this research are available from https:// itcproject.org/request-data-form/. In each country participating in the International Tobacco Control Policy Evaluation (ITC) Project, the data are jointly owned by the lead researcher(s) in that country and the ITC Project at the University of Waterloo. Data from the ITC Project are available to approved researchers two years after the date of issuance of cleaned data sets by the ITC Data Management Centre. Researchers interested in using ITC data are required to apply for approval by submitting an International Tobacco Control Data Repository (ITCDR) request application and subsequently signing an ITCDR Data Usage Agreement. The criteria for data usage approval and the contents of the Data Usage Agreement are described online (http://www.itcproject.org).

## Abbreviations

CI: confidence interval
ITC 4CV: International Tobacco Control 4 Country Smoking and Vaping
