# Supplementary material for: Awareness and use of a free vapes programme for smoking cessation in England: findings from the 2024 ITC 4 Country Smoking and Vaping Survey
Source: BMC Public Health. 2026 Aug 5;26:3340. doi: 10.1186/s12889-026-28769-0 (PMC13753483; doi:10.1186/s12889-026-28769-0)
Supplement: Supplementary file 1 [file 12889_2026_28769_MOESM1_ESM.docx]

**Supplemental Materials**

**Title:** Awareness and use of a free vapes programme for smoking cessation in England: Findings from the 2024 ITC 4 Country Smoking and Vaping Survey

**Authors:** Martine Skumlien PhD^1,2^, Katherine A East^1,3^, Erikas Simonavičius^4^, Andrew Hyland^5^, Geoffrey T Fong^6,7,8^, Grace Li^6^, Anne C K Quah^6^, Christian Boudreau^9^, Ann McNeill^1^, Leonie Brose^1^

**Affiliations:**

^1^Department of Addictions, Institute of Psychiatry, Psychology & Neuroscience, King’s College London, London, UK

^2^Social, Genetic & Developmental Psychiatry Centre, Institute of Psychiatry, Psychology & Neuroscience, King’s College London, London, UK

^3^ Department of Primary Care and Public Health, Brighton and Sussex Medical School, University of Brighton and University of Sussex, Brighton, UK

^4^European Union Drugs Agency, Lisbon, Portugal

^5^Roswell Park Comprehensive Cancer Centre, University of Buffalo, New York, USA

^6^Department of Psychology, University of Waterloo, Waterloo, Ontario, Canada

^7^School of Public Health Sciences, University of Waterloo, Waterloo, Ontario, Canada

^8^Ontario Institute for Cancer Research, Toronto, Ontario, Canada

^9^Department of Statistics and Actuarial Science, University of Waterloo, Waterloo, Ontario, Canada

# **Deviations from the pre-registered analysis protocol**

The current analyses were pre-registered to the Open Science Framework (https://osf.io/7x6ba). As we published the analyses prior to accessing the data, there were some deviations between the current and the pre-registered analyses.

First, we had to collapse some categories for Gender, Ethnicity, Income, and Vaping Status, due to there being too few participants in some of the sub-categories for these variables (see Supplemental Table 1). For Gender, we included other gender with women, as both groups are historically and typically disadvantaged relative to men. This was seen as preferable to excluding participants who identified as other gender. There were only seven people (0.3%) reporting other gender in the full sample, and it is therefore extremely unlikely that merging this group significantly biased our analyses. We also had to collapse Swap to Stop Interest and Swap to Stop Participation in the logistic regressions for the same reason. We noted that it might be necessary to collapse groups in the pre-registered analysis plan.

Second, we stated in our analysis protocol that we would include ‘refused’ or missing responses as a separate category if these resulted in >5% data loss. Of the participants who reported being aware of Swap to Stop and comprised the analytical sample for Swap to Stop Offer and Interest/Participation, 5.3% and 5.1% refused to answer the questions about mental and physical health diagnoses, respectively (Table 1). However, as the proportions of ‘refused’ responses for these questions in the total sample were 2.5% and 2.1%, respectively, and ‘refused’ responses for all other questions in all other models were ≤5%, we excluded these responses to preserve consistency across our analyses.

Third, we initially planned to conduct logistic regressions with Smoking Status (currently smoking daily, currently smoking non-daily, recently quit smoking) as a predictor. Given that people who do and do not smoke represent distinct populations, we first explored interactions between Smoking Status and the other predictors for each of our outcomes. Several of the interactions were statistically significant, suggesting that our planned models, which assumed that any effect of the other predictors would be equivalent in people who do and do not smoke, would not be valid. There were too few participants to include the interactions or to split models by Smoking Status. In other words, it was not possible to run valid models with this predictor, and we therefore decided to omit these models completely. However, we did run our planned logistic regressions with Past-Year Daily Smoking, which is similar to Smoking Status but which did not interact significantly with most of the other predictors in the logistic regressions.

Finally, we also stated in our protocol that we would descriptively explore overall smoking and quitting measures in the population, such as quit attempts, quit success, and use of efficacious treatments. However, as it was beyond the scope of the study to measure the *impact* of Swap to Stop on these outcomes, and because the current paper already included several outcomes and models, we decided to instead explore these questions in a future project.

# **Supplemental Tables**

**Supplemental Table 1.** Variable measurement and manipulation.

| Variable | Questions | Original response options | Manipulated response options (original responses) |
| --- | --- | --- | --- |
| SWAP TO STOP QUESTIONS |  |  |  |
| Swap to Stop Awareness | 'Swap to Stop' is a program to help people stop smoking. It gives participants a free e-cigarette (vape) starter kit and behavioural support.  Have you heard of Swap to Stop? | 1 Yes  2 No  8 Refused  9 Don't know | 0 No/don’t know (2, 9)  1 Yes (1)  NA (8) |
| Swap to Stop Offer | Has Swap to Stop been offered to you? | 1 Yes and I have participated  2 Yes but I did not participate  3 No it was not offered but I would be interested in participating  4 No it was not offered and I would not be interested in participating  8 Refused  9 Don't know | 0 No, it was not offered/don’t know (3, 4, 9)  1 Yes, it was offered (1, 2)  NA (8) |
| Swap to Stop Interest | Has Swap to Stop been offered to you? | 1 Yes and I have participated  2 Yes but I did not participate  3 No it was not offered but I would be interested in participating  4 No it was not offered and I would not be interested in participating  8 Refused  9 Don't know | 0 No, not interested in participating /don’t know (4, 9)  1 Yes, interested in participating (3)  NA (1, 2, 8) |
| Swap to Stop Participation | Has Swap to Stop been offered to you? | 1 Yes and I have participated  2 Yes but I did not participate  3 No it was not offered but I would be interested in participating  4 No it was not offered and I would not be interested in participating  8 Refused  9 Don't know | 0 No, did not participate /don’t know (2, 9)  1 Yes, participated (1)  NA (3, 4, 8) |
| Swap to Stop Interest/Participation | Has Swap to Stop been offered to you? | 1 Yes and I have participated  2 Yes but I did not participate  3 No it was not offered but I would be interested in participating  4 No it was not offered and I would not be interested in participating  8 Refused  9 Don't know | 0 No, did not participate/not interested in participating /don’t know (2, 4, 9)  1 Yes, participated/interested in participating (1, 3)  NA (8) |
| Reasons for not participating in Swap to Stop | Why didn’t you participate in Swap to Stop? / Why don’t you want to participate in Swap to Stop? | 1 Yes  2 No  8 Refused  9 Don't know  To the following statements:   - I don’t believe e-cigarettes will help me stop smoking - I don’t believe e-cigarettes are safe - I don’t believe e-cigarettes are less harmful than smoking - I don’t want support - I’m not interested in stopping smoking - I'm not interested in using e-cigarettes - There was a long waiting list - I didn’t like the e-cigarette(s) being offered - I can just buy e-cigarettes myself - Other | 0 No/don’t know (2, 9)  1 Yes (1)  NA (8) |
| Using Swap to Stop vape | Are you currently using the same type of e-cigarette (vape) that you were given as part of Swap to Stop? | 1 Yes  2 No  8 Refused  9 Don't know^a^ | 0 No (2)  1 Yes (1)  NA (8) |
| Had additional Swap to Stop support | Have you had any other support as part of Swap to Stop? This could for example be in-person meetings, phone calls or an app. | 1 Yes  2 No  8 Refused  9 Don't know^a^ | 0 No (2)  1 Yes (1)  NA (8) |
| How helpful was Swap to Stop | How helpful are you finding (did you find) Swap to Stop? | 1 Very helpful  2 Helpful  3 Neither helpful nor unhelpful  4 Unhelpful  5 Very unhelpful  8 Refused  9 Don't know^a^ | 0 Unhelpful (4, 5)  1 Neither helpful nor unhelpful (3)  2 Helpful (1, 2)  NA (8) |
| When started Swap to Stop | When did you start participating in Swap to Stop? | 1 Less than 1 month ago  2 1-3 months ago  3 4-6 months ago  4 Over 6 months ago  8 Refused  9 Don't know^a^ | 0 Less than 1 month ago (1)  1 1-6 months ago (2, 3)  2 Over 6 months ago (4)  NA (8) |
| SOCIODEMOGRAPHICS |  |  |  |
| Age-Group | *Derived variable – current age (categories)* | 1 18-24 years  2 25-39 years  3 40-54 years  4 55 years and up | 0 <25 years (1)  1 25-39 years (2)  2 40 years and over (3, 4) |
| Gender | *Derived variable, inclusive of non-binary and genderqueer gender identities/expressions* | 1 Man  2 Woman  3 Other  8 Refused  9 Don't know^a^ | 0 Man (1)  1 Woman or other (2)  Missing (8) |
| Ethnicity | Which of the following best describes your ethnic or racial background? | ~ White  01 English/ Welsh/ Scottish/ Northern Irish/ British  02 Irish  03 Gypsy or Irish Traveller  04 Any other White background  ~ Mixed/multiple ethnic groups  05 White and Black Caribbean  06 White and Black African  07 White and Asian  08 Any other Mixed/ multiple ethnic background  ~ Asian/Asian British  09 Indian  10 Pakistani  11 Bangladeshi  12 Chinese  13 Any other Asian background  ~ Black/African/Caribbean/Black British  14 African  15 Caribbean  16 Any other Black/ African/ Caribbean background  ~ Other ethnic group  17 Arab  18 Any other ethnic background  88 Refused  99 Don't know | 0 White/don’t know (01-04, 99)  1 Any other race/ethnicity (05-18)  NA (88) |
| Income | Which of the following categories best describes your ANNUAL household income, that is the total income before taxes, or gross income, of all persons in your household combined, for one year? | 01 Under £6,500  02 £6,500-15,000  03 £15,001-30,000  04 £30,001-40,000  05 £40,001-50,000  06 £50,001-65,000  07 £65,001-95,000  08 £95,001 and over  09 £6,500-10,000  10 £10,001-15,000  11 £15,001-20,000  12 £20,001-25,000  13 £25,001-30,000  14 £30,001-35,000  15 £35,001-40,000  16 £40,001-50,000  17 £50,001-65,000  18 £65,001-95,000  19 £95,001 and over  88 Refused  99 Don't know | 0 ≤£30,000/don’t know (01-03, 09-13, 99)  1 £30,001-£50,000 (04-05, 14-16)  2 ≥£50,001 (06-08, 17-19)  NA (88) |
| HEALTH |  |  |  |
| Mental Health Diagnosis | Have you ever been told by a doctor that you have a mental health condition, such as depression, anxiety, problematic substance use/substance use disorder, or other mental health condition? | 1 Yes  2 No  8 Refused  9 Don't know | 0 No/don’t know (2)  1 Yes (1)  NA (8) |
| Physical Health Diagnosis | Have you ever been diagnosed with any of the following health conditions: diabetes, heart disease, cancer, asthma, emphysema, or chronic bronchitis? | 1 Yes  2 No  8 Refused  9 Don't know | 0 No/don’t know (2)  1 Yes (1)  NA (8) |
| SMOKING AND VAPING |  |  |  |
| Cigarette Smoking Status | *Derived variable* | 10 Current daily smoker  20 Current weekly smoker  31 Current monthly smoker  32 Current less-than-monthly smoker  40 Recent Quitter: identifies as quit in last 12 months | 0 Recently quit smoking (40)  1 Currently smoking, non-daily (20, 31, 32)  2 Currently smoking, daily (10) |
| Past-Year Daily Smoking | *Derived variable – smoked daily at any point in the past year* | 0 Not smoked daily in the past 12 months/don’t know  1 Currently smoking daily/smoked daily in the past 12 months | 0 No/don’t know (0)  1 Yes (1) |
| Quit Attempt, ever |  | 1 Yes  2 No  8 Refused  9 Don't know | 0 No/don’t know (2)  1 Yes (1)  NA (8) |
| Vaping Status | *Derived variable* | 10 Current Daily Vaper  20 Current Weekly Vaper  31 Current Monthly Vaper  32 Current Less-than-monthly Vaper  40 Ever Quitter: past vaper at least weekly  50 Trier: vaped more than once/occasionally  60 Trier: vaped only once  70 Never Tried e-cigarettes  80 Never Heard of e-cigarettes | 0 Not currently vaping (40-80)  1 Currently vaping (10- 32) |
| Vaping harm perception | Compared to smoking cigarettes, how harmful do you think vaping is?  Vaping is… | 1 Much less harmful than smoking cigarettes  2 Somewhat less harmful  3 Equally harmful  4 Somewhat more harmful  5 Much more harmful  8 Refused  9 Don't know | 0 Equally or somewhat/much more harmful than smoking/don’t know (3, 4, 5, 9)  1 Much/somewhat less harmful than smoking (1, 2)  NA (8) |

^a^No participants responded ‘don’t know’ to this question.

**Supplemental Table 2.** Responses to additional Swap to Stop questions.

|  | ***n*** | **% (weighted)** | **95% confidence interval (weighted)** |
| --- | --- | --- | --- |
| **Using Swap to Stop vape^a^** | |  |  |
| Yes | 132 | 65.3 | 41.9 – 88.6 |
| No | 23 | 34.7 | 11.4 – 58.1 |
| **Had additional Swap to Stop support^b^** | |  |  |
| Yes | 131 | 69.4 | 46.9 – 91.9 |
| No | 29 | 30.6 | 8.1 – 53.2 |
| **How helpful was Swap to Stop^b^** | |  |  |
| Helpful | 153 | 96.2 | 89.9 – 98.6* |
| Neither helpful nor unhelpful | 4 | 2.2 | 0.6 – 7.4* |
| Unhelpful | 3 | 1.6 | 0.3 – 7.2* |
| **When started Swap to Stop^c^** | |  |  |
| Over 6 months ago | 7 | 3.9 | 1.3 – 11.5* |
| 1-6 months ago | 113 | 80.3 | 69.7 –90.8 |
| Less than 1 month ago | 41 | 15.8 | 6.9 – 24.7 |

^a^*n* = 155

^b^*n* = 160

^c^*n* = 161

*Confidence interval was computed using a logit transformation.

**Supplemental Table 3.** Results of unweighted binary multivariable logistic regression models.

|  | **Swap to Stop Awareness^a^** | | **Swap to Stop Offer^b^** | | **Swap to Stop Interest/Participation^b^** | | **Perceiving vaping to less harmful than smoking^c^** | | **Perceiving vaping to be less harmful than smoking, among those aware of Swap to Stop^d^** | |
| --- | --- | --- | --- | --- | --- | --- | --- | --- | --- | --- |
|  | **AOR (95% CI)** | ***p*** | **AOR (95% CI)** | ***p*** | **AOR (95% CI)** | ***p*** | **AOR (95% CI)** | ***p*** | **AOR (95% CI)** | ***p*** |
| **Age Group** |  |  |  |  |  |  |  |  |  |  |
| ≤24 years (ref) | - | - | - | - | - | - | - | - | - | - |
| 25-39 years | **0.56**  **(0.36 – 0.87)** | **.01** | **0.34**  **(0.14 – 0.76)** | **.01** | **0.45**  **(0.23 – 0.89)** | **.02** | 0.17  (0.79 – 1.74) | .44 | 0.69  (0.34 – 1.39) | .31 |
| ≥40 years | **0.12**  **(0.08- 0.19)** | **<.001** | **0.10**  **(0.04 – 0.24)** | **<.001** | 0.69  (0.32 – 1.47) | .34 | **1.95**  **(1.33 – 2.88)** | **.001** | 0.85  (0.40 – 1.83) | .68 |
| **Gender** |  |  |  |  |  |  |  |  |  |  |
| Man (ref) | - | - | - | - | - | - | - | - | - | - |
| Woman or Other | **0.66**  **(0.50 – 0.88)** | **.005** | 0.81  (0.50 – 1.34) | .41 | 0.95  (0.59 – 1.52) | .82 | **0.80**  **(0.65 – 0.98)** | **.03** | 1.37  (0.84 – 2.24) | .21 |
| **Ethnicity** |  |  |  |  |  |  |  |  |  |  |
| White (ref) | - | - | - | - | - | - | - | - | - | - |
| Any other race/ethnicity | 1.13  (0.78 – 1.64) | .51 | **0.52**  **(0.29 – 0.92)** | **.03** | 0.80  (0.46 – 1.42) | .44 | 1.07  (0.79 – 1.43) | .67 | 1.55  (0.86 – 2.83) | .15 |
| **Income** |  |  |  |  |  |  |  |  |  |  |
| ≤£30,000 (ref) | - | - | - | - | - | - | - | - | - | - |
| £30,001 - £50,000 | **1.62**  **(1.12 – 2.36)** | **.01** | 0.80  (0.40 – 1.61) | .54 | 1.30  (0.70 – 2.43) | .40 | **1.57**  **(1.22 – 2.02)** | **<.001** | **4.48**  **(2.36 – 8.72)** | **<.001** |
| ≥£50,001 | **2.22**  **(1.55 – 3.19)** | **<.001** | 0.65  (0.33 – 1.26) | .21 | 1.48  (0.81 – 2.72) | .21 | **1.58**  **(1.23 – 2.05)** | **<.001** | **4.08**  **(2.13 – 8.04)** | **<.001** |
| **Mental Health Diagnosis** | |  |  |  |  |  |  |  |  |  |
| No (ref) | - | - | - | - | - | - | - | - | - | - |
| Yes | 1.20  (0.84 – 1.72) | .32 | 0.63  (0.31 – 1.28) | .20 | 0.87  (0.46 – 1.64) | .66 | 1.15  (0.90 – 1.47) | .25 | 0.72  (0.37 – 1.41) | .34 |
| **Physical Health Diagnosis** | |  |  |  |  |  |  |  |  |  |
| No (ref) | - | - | - | - | - | - | - | - | - | - |
| Yes | 1.31  (0.90 – 1.91) | .16 | **2.52**  **(1.15 – 5.76)** | **.02** | 1.18  (0.60 – 2.35) | .64 | 0.79  (0.61 – 1.01) | .06 | 0.59  (0.28 – 1.21) | .15 |
| **Past-year Daily Smoking** | |  |  |  |  |  |  |  |  |  |
| No (ref) | - | - | - | - | - | - | - | - | - | - |
| Yes | **1.52**  **(1.06 – 2.21)** | **.03** | 1.44  (0.79 – 2.63) | .23 | 1.14  (0.62 – 2.05) | .68 | 0.88  (0.67 – 1.16) | .37 | 0.74  (0.40 – 1.36) | .34 |
| **Quit Attempts** |  |  |  |  |  |  |  |  |  |  |
| No (ref) | - | - | - | - | - | - | - | - | - | - |
| Yes | **1.90**  **(1.40 – 2.58)** | **<.001** | 1.09  (0.65 – 1.82) | .74 | **1.81**  **(1.11 – 2.98)** | **.02** | **1.73**  **(1.39 – 2.15)** | **<.001** | **1.72**  **(1.03 – 2.90)** | **.04** |
| **Vaping Status** |  |  |  |  |  |  |  |  |  |  |
| Not currently vaping (ref) | - | - | - | - | - | - | - | - | - | - |
| Currently vaping | **4.39**  **(3.02 – 6.53)** | **<.001** | **8.59 (3.59 – 23.24)** | **<.001** | **3.68**  **(1.77 – 7.89)** | **.001** | **2.57**  **(2.05 – 3.23)** | **<.001** | **2.35**  **(1.03 – 5.72)** | **.05** |
| **Swap to Stop awareness** |  |  |  |  |  |  |  |  |  |  |
| No (ref) | - | - | - | - | - | - | - | - | - | - |
| Yes | - | - | - | - | - | - | 0.99  (0.76 – 1.29) | .96 | - | - |
| **Swap to Stop interest or participation** |  |  |  |  |  |  |  |  |  |  |
| No (ref) | - | - | - | - | - | - | - | - | - | **-** |
| Yes | - | - | - | - | - | - | - | - | **1.68**  **(1.04 – 2.72)** | **.03** |

^a^*n* = 1844

^b^*n* = 396

^c^*n* = 1829

^d^*n* = 393

*Abbreviations: AOR,* adjusted odds ratio; *CI,* confidence interval; *ref*, reference category.

*Note*. A Wald 2 d.f. test showed that the overall effect of Age Group was significant for Awareness (*F* = 61.99, *p* < .001), Offer (*F* = 16.49, *p* < .001), and Interest/Participation (*F* = 3.03, *p* = .05). Odds ratios for the Age Group comparison ≥40 years versus 25-39 years were 0.22 (0.16 – 0.30) for Awareness, 0.30 (0.18 – 0.52) for Offer, and 1.52 (0.88 – 2.64) for Interest/Participation. A Wald 2 d.f. test showed that the overall effect of Income was significant for Awareness (*F* = 9.40, *p* < .001) but not for Offer (*F* = 0.81, *p* = .44), or Interest/Participation (*F* = 0.82, *p* = .44). Odds ratios for the Income comparison ≥£51,001 versus £30,001-£50,000 were 1.37 (0.95 – 1.96) for Awareness, 0.81 (0.44 – 1.49) for Offer, and 1.14 (0.63 – 2.05) for Interest/Participation.
